# A treatment-planning comparison of three beam arrangement strategies for stereotactic body radiation therapy for centrally located lung tumors using volumetric-modulated arc therapy

**DOI:** 10.1093/jrr/rrv105

**Published:** 2016-06-21

**Authors:** Kentaro Ishii, Wataru Okada, Ryo Ogino, Kazuki Kubo, Shun Kishimoto, Ryuta Nakahara, Ryu Kawamorita, Yoshie Ishii, Takuhito Tada, Toshifumi Nakajima

**Affiliations:** 1 Department of Radiation Oncology, Tane General Hospital, 1-12-21 Kujo-minami, Nishi-ku, Osaka, 550-0025, Japan; 2 Department of Radiation Oncology, Yodogawa Christian Hospital, 1-7-50 Kunijima, Higashiyodogawa-ku, Osaka, 533-0024, Japan; 3 Department of Radiology, Izumi Municipal Hospital, 4-10-10 Futyu-cho, Izumi, 594-0071, Japan

**Keywords:** lung tumor, stereotactic body radiation therapy, volumetric-modulated arc therapy, planning study

## Abstract

The purpose of this study was to determine appropriate beam arrangement for volumetric-modulated arc therapy (VMAT)-based stereotactic body radiation therapy (SBRT) in the treatment of patients with centrally located lung tumors. Fifteen consecutive patients with centrally located lung tumors treated at our institution were enrolled. For each patient, three VMAT plans were generated using two coplanar partial arcs (CP VMAT), two non-coplanar partial arcs (NCP VMAT), and one coplanar full arc (Full VMAT). All plans were designed to deliver 70 Gy in 10 fractions. Target coverage and sparing of organs at risk (OARs) were compared across techniques. PTV coverage was almost identical for all approaches. The whole lung V_10Gy_ was significantly lower with CP VMAT plans than with NCP VMAT plans, whereas no significant differences in the mean lung dose, V_5Gy_, V_20Gy_ or V_40Gy_ were observed. Full VMAT increased mean contralateral lung V_5Gy_ by 12.57% and 9.15% when compared with NCP VMAT and CP VMAT, respectively. Although NCP VMAT plans best achieved the dose–volume constraints for mediastinal OARs, the absolute differences in dose were small when compared with CP VMAT. These results suggest that partial-arc VMAT may be preferable to minimize unnecessary exposure to the contralateral lung, and use of NCP VMAT should be considered when the dose–volume constraints are not achieved by CP VMAT.

## INTRODUCTION

Stereotactic body radiation therapy (SBRT) has proven to be an effective treatment modality for medically inoperable early-stage non–small cell lung cancer (NSCLC) and lung metastases [1, 2]. However, the favorable data on lung SBRT are based mainly on the treatment of peripheral lesions. The use of SBRT for centrally located lung tumors still remains controversial due to the potential of severe toxicity, such as bronchial stenosis, hemoptysis, and fistulas [3]. Therefore, reducing the radiation dose to organs at risk (OARs) in the mediastinum and pulmonary hilum is essential when treating centrally located lung lesions with SBRT.

Volumetric-modulated arc therapy (VMAT) is now widely used clinically because it can deliver intensity-modulated radiation therapy (IMRT) quality plans with superior treatment delivery efficiency. As for lung SBRT, several treatment planning studies have shown that VMAT achieved superior dose conformity and OAR sparing with a shorter treatment time than 3D conformal radiotherapy (3D-CRT) [4–8] or conventional IMRT [9, 10]. With these promising results, there has been an increase in the use of VMAT in lung SBRT. A patterns-of-care survey for lung SBRT performed in 2012 reported that 47% of radiation oncologists have used rotational IMRT in their practice in the USA [11].

The treatment beam arrangements used for lung SBRT are either coplanar, non-coplanar or a mixture of both. Many institutions use multiple non-coplanar beams for lung SBRT using 3D-CRT [12, 13]. A study comparing beam arrangements for 3D-CRT-based lung SBRT concluded that non-coplanar beams were best when compared with coplanar beams or arcs (volumes at high doses were equivalent between all, but volumes at low doses were better with non-coplanar beams) [14]. VMAT has the ability for non-coplanar arc delivery, but the current implementations of VMAT have focused on coplanar delivery [4–6, 9, 10, 15–19]. In addition, the range of arc used for VMAT-based lung SBRT varies among institutions: some institutions use full arc [4, 6, 8, 10, 16, 17] and others use partial arc to avoid the contralateral lung [5, 9, 15, 16, 18]. However, data for optimal beam arrangements are limited to date.

This study was undertaken to determine appropriate beam arrangement for VMAT-based SBRT in the treatment of patients with centrally located lung tumors that require better OAR sparing when compared with patients with peripheral lesions.

## MATERIALS AND METHODS

### Patient selection and contouring

This study was approved by the institutional ethics committee, and written informed consent was obtained from all patients. Computed tomography (CT) datasets of 15 consecutive patients with centrally located lung tumors who were treated with VMAT-based SBRT in our institution between November 2011 and February 2015 were included in this study. Central location was defined as the area within 2 cm of critical structures, such as the bronchial tree, great vessels, esophagus, and heart, but without direct involvement of the mediastinal structures. Those OARs located within 2 cm of the tumor were defined as immediately adjacent OARs.

Four-dimensional CT images were acquired in the supine position, with 1.25-mm slice thickness after immobilization with the BodyFix double vacuum immobilization system (Elekta, Stockholm, Sweden). Scans were performed using the Varian Real-time Position Management (RPM) system (Varian Medical Systems, Palo Alto, CA). The gross tumor volume (GTV) was contoured at the lung window level on all 10 respiratory phase CT datasets. These 10 GTVs were fused to form the internal target volume (ITV). The planning target volume (PTV) was generated by adding a 5-mm margin to the ITV in all dimensions to account for set-up errors. Contouring of OARs, including the whole lung, esophagus, heart, spinal cord, bronchial tree, and great vessels (aorta, vena cavae, pulmonary artery, and pulmonary vein), was defined according to the Radiation Therapy Oncology Group (RTOG) protocol 0813 [20]. The bronchial tree consisted of the trachea and the proximal bronchial tree. The contralateral lung was also contoured as a separate structure. Dose constraints used for OARs are listed in Table 1 and were based primarily on the MD Anderson Cancer Center experiences using 70 Gy in 10 fractions [21].

**Table 1.** Dose–volume constraints to OARs used for plan evaluation

Structure	Dose–volume constraints
Whole lung	MLD ≤ 9 Gy
	V_40Gy_ ≤ 7%
Esophagus	V_40Gy_ ≤ 1 cm^3^
Heart	D_0.1cm^3^_ ≤ 60 Gy
	V_45Gy_ ≤ 1 cm^3^
Spinal cord	D_0.1cm^3^_ ≤ 30 Gy
Bronchial tree	D_0.1cm^3^_ ≤ 60 Gy
	V_50Gy_ ≤ 1 cm^3^
Great vessels	D_0.1cm^3^_ ≤ 75 Gy
	V_50Gy_ ≤ 1 cm^3^

OARs = organs at risk, MLD = mean lung dose, V*_n_*_Gy_ = percentage or absolute structure volume receiving ≥*n* Gy, D_0.1cm^3^_ = maximal dose received by 0.1 cm^3^ to the structure.

### Treatment planning

**Fig. 1.**
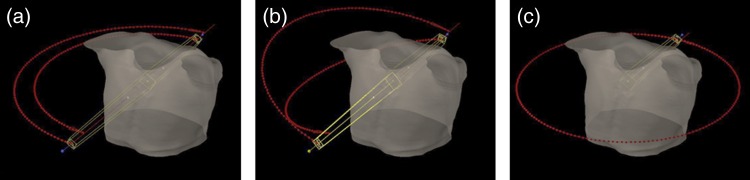
Representative beam arrangement for (**a**) two coplanar partial arcs volumetric-modulated arc therapy (VMAT), (**b**) two noncoplanar partial arcs VMAT and (**c**) one coplanar full arc VMAT.

All plans prescribed 70 Gy delivered in 10 daily fractions to the PTV. They were normalized so that at least 95% of the PTV received the prescribed dose. Three VMAT plans were generated with two coplanar partial arcs (CP VMAT), two non-coplanar partial arcs (NCP VMAT) and one coplanar full arc (Full VMAT). CP VMAT and NCP VMAT plans consisted of two ipsilateral arcs of 179° with collimator angles of 30° and 330°, and Full VMAT plans consisted of one arc of 358° with a collimator angle of 30°. The couch was set at ± 15° to the straight position for the NCP VMAT plans. The arrangement of the fields for a representative patient is shown in Fig. 1. Mean field sizes by jaws in CP VMAT, NCP VMAT, and Full VMAT were 5.7 × 5.4 cm^2^, 5.6 × 5.5 cm^2^, and 5.9 × 5.6 cm^2^, respectively, where results were reported as the average of the two arcs for CP VMAT and NCP VMAT. All VMAT plans were developed in the Eclipse v.10.0 treatment planning system using 6-MV photons in a Novalis Tx treatment unit (Varian Medical Systems, and BrainLAB AG, Feldkirchen, Germany). The same optimization template was utilized for the three VMAT techniques (Table 2); however, these objectives were modified during the optimization process based on the real-time updated dose–volume histograms (DVHs) of the PTV and OARs. To achieve the highly steep gradient dose to the PTV, we used the normal tissue objective (a parameter that limits dose as a function of distance from the outer border of the PTV) with a priority of 150 and a 0.15 fall-off between the start dose of 105% and the end dose of 60%. In addition, avoidance structures were used for optimization to minimize the dose to OARs. The optimization constraints and priorities of these avoidance structures were not initially applied and were real-time adjusted during the progression of optimization. During the optimization process, the primary goal was to achieve similar PTV coverage for the three VMAT plans, and the secondary goal was to decrease the OAR doses as much as possible. Multiple plans for each VMAT technique were generated as we performed optimization several times, further tightening dose–volume constraints of the OARs each time, and the most preferable plan was selected for a fair comparison. The final dose calculation was performed using the anisotropic analytical algorithm with a 1.25-mm grid resolution.

**Table 2.** Initial optimization parameters

Structure	Limit	Volume (%)	Dose (Gy)	Priority
PTV	upper	0	72	100
	lower	95	70	130
	lower	100	69	130
Lungs minus PTV	upper	5	40	80
	upper	10	20	80
Contralateral lung	upper	0	20	80
Esophagus	upper	0	35	80
Heart	upper	0	50	80
Spinal cord	upper	0	20	80
Bronchial tree	upper	0	50	80
Great vessels	upper	0	65	80

PTV = planning target volume.

### Plan evaluation

DVHs for the PTV, whole lung, contralateral lung, esophagus, heart, spinal cord, bronchial tree, and great vessels were calculated for each subject. Parameters chosen for comparison were mean dose, D_2%_, and D_98%_ for the PTV, where D*_n_*_%_ is the minimal dose delivered to *n*% of the PTV. The homogeneity index (HI) and conformation number (CN) for the PTV were calculated as below [15, 22]:

$$\text{HI }=\;({\text{D}}_{2\%}-\;{\text{D}}_{98\%})/{{\text{D}}_{p}}_{\;}\times \;100\%$$

CN= (PTV encompassed by 95% isodose/PTV)×(PTV encompassed by 95% isodose/95% isodose volume),

where D*_p_* is the prescription dose. For the CN, the first portion is an assessment of target volume coverage by 95% of the prescription dose, and the second portion is an assessment of normal tissue sparing. The CN values range between 0 and 1, with 1 representing the ideal conformity. Mean lung dose, V_5Gy_, V_10Gy_, V_20Gy_ and V_40Gy_ for the whole lung and V_5Gy_ for the contralateral lung were compared, where V*_n_*_Gy_ is the percentage structure volume receiving at least *n* Gy. The maximal dose received by 0.1 cm^3^ (D_0.1cm^3^_) of the esophagus, heart, spinal cord, bronchial tree, and great vessels was also compared. The number of Monitor Units (MU) needed for each plan was collected to determine the dose-delivery efficiency of each treatment. Patients were divided into two groups based on whether all VMAT techniques met the dose–volume constraints (Group 1) or not (Group 2). The distance from the PTV to its immediately adjacent OAR was recorded for each patient.

### Statistical analysis

Dose–volume parameters, HI, CN and MU were compared by Wilcoxon matched-pair signed-rank tests for non-parametric data. The Mann–Whitney *U* test was used to analyze the difference in the closest distance to the PTV between Groups 1 and 2. Significance was defined at *P* < 0.05. All reported *P*-values are two-tailed.

## RESULTS

**Fig. 2.**
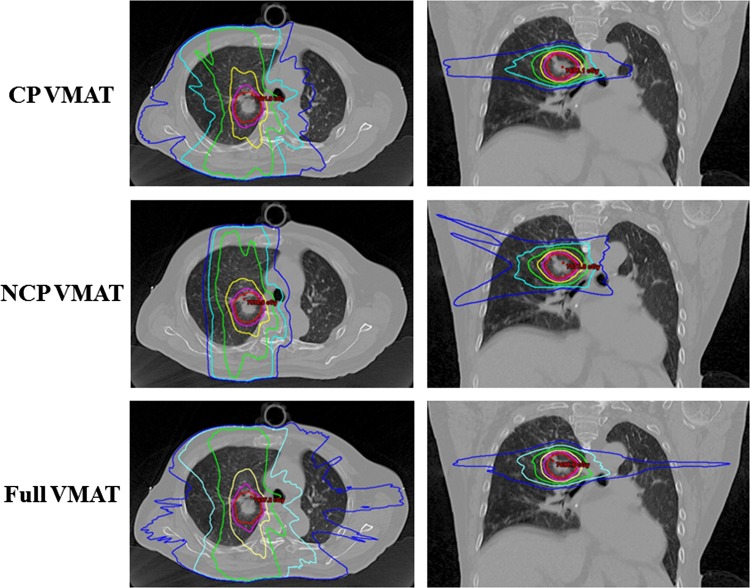
Dose distributions for two coplanar arcs volumetric-modulated arc therapy (CP VMAT), two noncoplanar partial arcs VMAT (NCP VMAT), and one coplanar full arc VMAT (Full VMAT) for the example case shown in Fig. 1. Planning target volume (PTV) is shown in pink. The following isodose lines are shown: 70 Gy (red), 60 Gy (purple), 40 Gy (yellow), 20 Gy (green), 10 Gy (cyan) and 5 Gy (blue).

The characteristics of the 15 patients are listed in Table 3. The comparison of dose distributions is illustrated in Fig. 2 for the example case shown in Fig. 1 for each of the three VMAT techniques. A comparison of mean values of the dosimetric parameters of the PTV and the OARs is provided in Table 4.

**Table 3.** Patient characteristics (*n* = 15)

Characteristic	Value
Age (y)	
Median	80
Range	66–91
PTV (cm^3^)	
Mean	46.4
Range	9.4–92.8
Tumor location (*n*)	
Left lung	10
Upper lobe	5
Lower lobe	5
Right lung	5
Upper lobe	3
Middle lobe	1
Lower lobe	1
Immediately adjacent structure (*n*)	
Esophagus	2
Heart	5
Spinal cord	0
Bronchial tree	6
Aorta	9
Vena cavae	2
Pulmonary artery	4
Pulmonary vein	1

PTV = planning target volume.

**Table 4.** Comparison of mean values of the dosimetric parameters of the PTV and the OARs

Parameter		Treatment technique			*P*-value		
		CP VMAT (mean ± SD)	NCP VMAT (mean ± SD)	Full VMAT (mean ± SD)	CP VMAT vs NCP VMAT	CP VMAT vs Full VMAT	NCP VMAT vs Full VMAT
PTV	D_mean_ (Gy)	72.33 ± 0.37	72.42 ± 0.37	72.40 ± 0.44	0.118	0.096	0.691
	D_2%_ (Gy)	74.56 ± 0.57	74.58 ± 0.59	74.60 ± 0.62	0.470	0.470	0.910
	D_98%_ (Gy)	69.20 ± 0.16	69.15 ± 0.17	69.16 ± 0.16	0.244	0.427	0.701
	HI	7.25 ± 0.91	7.34 ± 0.94	7.34 ± 0.99	0.427	0.256	0.875
	CN	0.72 ± 0.05	0.72 ± 0.05	0.71 ± 0.05	0.460	0.156	0.363
Whole lung	MLD (Gy)	5.26 ± 1.27	5.34 ± 1.35	5.63 ± 1.41	0.233	<0.001^a^	0.002^a^
	V_5Gy_ (%)	18.53 ± 5.57	19.37 ± 6.16	23.80 ± 7.06	0.256	<0.001^a^	0.015^a^
	V_10Gy_ (%)	12.81 ± 3.61	13.65 ± 4.15	13.39 ± 3.88	0.011^a^	0.015^a^	0.394
	V_20Gy_ (%)	8.36 ± 2.69	8.10 ± 2.63	8.51 ± 2.94	0.156	0.349	0.019^a^
	V_40Gy_ (%)	3.27 ± 1.15	3.20 ± 1.14	3.45 ± 1.35	0.244	0.078	0.044^a^
Contralateral lung	V_5Gy_ (%)	5.62 ± 6.27	2.20 ± 3.93	14.77 ± 9.96	<0.001^a^	<0.001^a^	<0.001^a^
Esophagus	D_0.1cm^3^_ (Gy)	13.61 ± 4.99	11.71 ± 4.79	14.05 ± 4.50	<0.001^a^	0.061	<0.001^a^
Heart	D_0.1cm^3^_ (Gy)	18.97 ± 19.20	17.81 ± 17.08	20.02 ± 20.30	0.307	0.041^a^	0.256
Spinal cord	D_0.1cm^3^_ (Gy)	10.65 ± 5.21	9.41 ± 5.01	11.49 ± 5.46	<0.001^a^	0.008^a^	<0.001^a^
Bronchial tree	D_0.1cm^3^_ (Gy)	14.86 ± 13.60	14.30 ± 12.17	15.81 ± 14.05	0.650	0.020^a^	0.047^a^
Aorta	D_0.1cm^3^_ (Gy)	35.89 ± 23.34	33.89 ± 22.74	36.60 ± 22.92	<0.001^a^	0.125	<0.001^a^
Vena cavae	D_0.1cm^3^_ (Gy)	10.45 ± 10.54	8.77 ± 10.15	11.31 ± 10.86	0.004^a^	0.011^a^	0.002^a^
Pulmonary artery	D_0.1cm^3^_ (Gy)	13.48 ± 15.33	13.79 ± 14.41	13.91 ± 15.30	0.307	0.069	0.842
Pulmonary vein	D_0.1cm^3^_ (Gy)	4.40 ± 4.95	4.60 ± 4.48	4.59 ± 5.54	0.433	0.087	0.594

PTV = planning target volume, OARs = organs at risk, CP VMAT = two coplanar partial arcs volumetric-modulated arc therapy, NCP VMAT = two non-coplanar partial arcs VMAT, Full VMAT = one coplanar full arc VMAT, SD = standard deviation, D_mean_ = mean dose to the PTV, D*_n_*_%_ = minimal dose to the percentage of the PTV, HI = homogeneity index, CN = conformation number, MLD = mean lung dose, V*_n_*_Gy_ = percentage structure volume receiving ≥*n* Gy, D_0.1cm^3^_ = maximal dose received by 0.1 cm^3^ to the structure. Two-sided *P*-values calculated using Wilcoxon matched-pair signed rank test. ^a^Significant at *P* ≤ 0.05.

The three VMAT techniques provided highly conformal dose distributions in terms of target coverage. All plans were almost identical with respect to the resulting dosimetric parameters of the PTV, HI and CN. The NCP VMAT and CP VMAT plans showed better sparing of the whole lung than the Full VMAT plans. The whole lung V_10Gy_ was significantly lower in the CP VMAT plans than in the NCP VMAT plans, whereas no significant differences were observed in the mean lung dose, V_5Gy_, V_20Gy_ or V_40Gy_. NCP VMAT plans achieved the best contralateral lung sparing. On the other hand, Full VMAT increased mean contralateral lung V_5Gy_ by 12.57% and 9.15% when compared with NCP VMAT and CP VMAT, respectively. NCP VMAT plans showed better sparing of mediastinal OARs than CP VMAT plans. Although NCP VMAT plans resulted in significantly lower D_0.1cm^3^_ to the esophagus, spinal cord, aorta, and vena cavae compared with CP VMAT plans, the absolute differences in mean dose were small: 1.9 Gy, 1.24 Gy, 2 Gy and 1.68 Gy, respectively. CP VMAT consistently provided equivocal or favorable mediastinal OAR sparing when compared with Full VMAT, although the difference was very small in absolute terms. The mean MUs for CP VMAT, NCP VMAT and Full VMAT were 1889 ± 197, 1850 ± 216 and 1832 ± 71, respectively, showing that there were no statistically significant differences between the three techniques.

All dose–volume constraints were met satisfactorily by the NCP VMAT plans for all patients. However, four immediately adjacent OARs in three patients and five immediately adjacent OARs in four patients failed to meet the dose–volume constraints by CP VMAT plans and Full VMAT plans, respectively. Table 5 shows a list of the dosimetric parameters of the immediately adjacent OARs that received more than the threshold value and the distance from the PTV to each OAR in Group 2 patients. The mean PTV to its closest OAR distance was significantly shorter in Group 2 than in Group 1 (0.13 cm vs 0.46 cm, *P* = 0.004). In 10 cases where the distance between the PTV and closest OAR was 0.25 cm or greater, optimal OAR sparing was achieved through all VMAT techniques. In cases where the distance was shorter than 0.25 cm, it was not possible to achieve optimal OAR sparing through CP VMAT, Full VMAT or both in four of five cases.

**Table 5.** Dosimetric parameters of the immediately adjacent OARs that received more than the threshold value and the distance from the PTV to each OAR in Group 2 patients

Patient	Immediately adjacent structures	PTV to structure distance (cm)	Parameter	Treatment technique		
				CP VMAT	NCP VMAT	Full VMAT
1	Aorta	0.10	V_50Gy_ (cm^3^)	0.83	0.75	1.01^a^
2	Aorta	0.10	V_50Gy_ (cm^3^)	1.32^a^	0.93	1.55^a^
	Heart	0.19	V_45Gy_ (cm^3^)	2.12^a^	0.76	2.65^a^
3	Heart	0.23	V_45Gy_ (cm^3^)	1.85^a^	0.68	4.27^a^
4	Aorta	0.10	V_50Gy_ (cm^3^)	1.96^a^	0.87	2.31^a^

OARs = organs at risk, PTV = planning target volume, CP VMAT = two coplanar partial arcs volumetric-modulated arc therapy, NCP VMAT = two non-coplanar partial arcs VMAT, Full VMAT = one coplanar full arc VMAT, V*_n_*_Gy_ = absolute structure volume receiving ≥*n* Gy. ^a^Values above the dose limit.

## DISCUSSION

VMAT has recently played an important role in SBRT of lung tumors. In the present study, appropriate beam arrangement for lung SBRT using VMAT was investigated. Since most OARs are removed from the high-dose area in the treatment of peripheral lung tumors, the dose constraints are easily met using 3D-CRT. Thus, there is controversy as to whether VMAT should be routinely used to treat peripheral lung tumors [23]. On the other hand, it is more challenging to generate an acceptable SBRT plan using 3D-CRT for centrally located lung tumors because of the close proximity of critical structures, which can be better handled with IMRT or VMAT. Therefore, 15 consecutive patients with centrally located tumors were selected for this dosimetric study. As for SBRT for centrally located lung cancer, most institutions have adopted more conservative fractionation schemes [21, 24, 25]. Recently, a report from the MD Anderson Cancer Center showed that SBRT with 70 Gy in 10 fractions achieved excellent local control and acceptable toxicity for clinically challenging cases, including centrally located lesions, compared with 50 Gy in 4 fractions [21]. In the current study, this dose fractionation schedule, as well as recommended dose–volume constraints, was followed. However, differing from the recommended dose–volume constraints, we used D_0.1cm^3^_ instead of maximum point dose, because maximum point doses are very sensitive to many factors such as dose calculation grid size and algorithm.

Several recent studies have compared VMAT with 3D-CRT for lung SBRT. In general, VMAT has shown better conformity to the PTV than 3D-CRT [4, 6–8]. The increase in the volume of normal lung that receives a low dose of radiation is a concern associated with VMAT. However, lung V_20Gy_/_12.5Gy_/_10Gy_/_5Gy_ values were significantly lower with VMAT than with 3D-CRT [5]. A recent study indicated that VMAT improved mean lung dose, lung V_5Gy_ and V_20Gy_ [7]. For the contralateral lung, Rauschenbach *et al*. showed a lower mean dose with VMAT than with 3D-CRT [8], whereas Ong *et al*. reported that VMAT led to a small increase in V_5Gy_ [6]. Therefore, the results of these studies showed that VMAT outperformed 3D-CRT in most dosimetric aspects. When compared with IMRT, coplanar VMAT achieved treatment plan qualities comparable with the non-coplanar IMRT technique and better than those of coplanar IMRT [9].

Many institutions have reported promising results with SBRT using VMAT in patients with lung tumors. However, there are very few data in the literature on dosimetric comparisons between various beam arrangements. To the best of our knowledge, there is only one planning study comparing coplanar VMAT with non-coplanar VMAT in the setting of lung SBRT. Zhang *et al*. showed that the dosimetric differences between coplanar and non-coplanar VMAT were not significant [7]. However, the angle separation of the non-coplanar arc was uniformly set at 10°. As they discussed, it was presumed that the dosimetric differences are expected to increase, favoring the non-coplanar VMAT plans, with a larger angle separation. In the present study, the couch was offset ± 15°; thus, the angle separation was set at 30°. This large angle separation may lead to better mediastinal OAR sparing compared with coplanar VMAT.

All three VMAT techniques produced highly conformal dose distributions around the PTV. Li *et al*. suggested that compromised PTV coverage contributed to local failure after lung SBRT [21]. With respect to PTV coverage, VMAT seems to be an appropriate technique for lung SBRT.

The use of Full VMAT, compared with CP VMAT or NCP VMAT, resulted in increased doses to most of the OARs. In particular, contralateral lung V_5Gy_ was markedly increased with Full VMAT. Ong *et al*. reported that contralateral lung V_5Gy_ strongly correlates with radiation pneumonitis after lung SBRT using VMAT [16]. In addition, Full VMAT offered only a small advantage over CP VMAT in terms of treatment time, because coplanar beams were used for both techniques. Therefore, it is conceivable that partial-arc VMAT is more suitable for lung SBRT to minimize unnecessary dose deposition to the contralateral lung.

Lung dosimetric parameters in CP VMAT were comparable with those of NCP VMAT: whole lung V_5Gy_, V_20Gy_, V_40Gy_ and mean lung dose were identical for the two techniques, whereas whole lung V_10Gy_ was better with CP VMAT, and contralateral lung V_5Gy_ was lower with NCP VMAT. Optimal OAR sparing was achieved in all cases by NCP VMAT, but in only 12 cases by CP VMAT. However, the changes in the quantitative dosimetric differences for mediastinal OARs were small. Furthermore, NCP VMAT generally requires more time to deliver treatments than CP VMAT due to the time required for positioning of the couch. The European Organisation for Research and Treatment of Cancer (EORTC) recommends coplanar beam arrangements for lung SBRT, as long as the dose distribution criteria are met, to keep the treatment time as short as possible [26]. With respect to this study, all CP VMAT plans fulfilled the dose requirements for OARs in all cases where the distance between the PTV and closest OAR was 0.25 cm or greater. Based on the present results, CP VMAT is sufficient for SBRT for centrally located lung tumors when all dose–volume constraints can be achieved. NCP VMAT may be more appropriate only in patients who require better mediastinal OAR sparing of OARs that are very close to the PTV.

This study had several limitations. In practice, the applicable area of the non-coplanar beam direction is limited in lung SBRT using VMAT due to a risk of collision between the couch and the gantry. In the present study, the angle separation of the non-coplanar arc was uniformly set at 30°, the maximum angle at our institution. However, it is likely that the angle separation is smaller depending on the linear accelerator and immobilization system used. In such cases, the benefit of using the non-coplanar beam becomes less. Additionally, evaluating the optimal number of beams is beyond the scope of this study. However, Chi *et al*. compared OAR dose parameters between 2 and 8-arc VMAT plans in delivering SBRT for centrally located lung tumors; they concluded that increasing the number of arcs in VMAT cannot significantly improve OAR sparing [15].

In conclusion, VMAT techniques provided highly conformal target coverage for SBRT for lung tumors. Considering the increase in dose to the contralateral lung without improvement in mediastinal OAR sparing with Full VMAT, beams that avoid the contralateral lung may be preferable. Although NCP VMAT plans best achieved the dose–volume constraints for mediastinal OARs for centrally located lung tumors, the absolute differences in doses were small when compared with CP VMAT. Therefore, we suggest that adoption of NCP VMAT be considered, in practice, when the dose–volume constraints are not achieved by CP VMAT due to shorter distances between the PTV and OARs.

## FUNDING

Funding to pay the Open Access publication charges for this article was provided by K.I. (Tane General Hospital).
